# Dr. John Thomson

**Published:** 1909-05-15

**Authors:** 


News has lately been received in London by telegram of the death of Dr. John Thomson, a prominent medical officer practising at Brisbane, Queensland, at the age of sixty-one. Dr. Thomson graduated in 1871 at the University of Edinburgh, and settled in Brisbane over thirty-three years ago. He identified himself chiefly with matters relating to sanitary science, on which he soon came to be recognised as an authority. He was President of the Royal Society of Queensland, and his colleagues elected him President of the Intercolonial Medical Congress in 1899. In addition to other appointments, he was an honorary associate of the Order of St. John of Jerusalem and colonel and principal medical officer of the Commonwealth military forces of Australia.

